# Photoregulation of PRMT-1 Using a Photolabile Non-Canonical Amino Acid

**DOI:** 10.3390/molecules26165072

**Published:** 2021-08-21

**Authors:** Elizabeth A. King, Emily M. Peairs, Diya M. Uthappa, Jordan K. Villa, Cameron M. Goff, Naya K. Burrow, Rebecca T. Deitch, Anna K. Martin, Douglas D. Young

**Affiliations:** Department of Chemistry, College of William & Mary, P.O. Box 8795, Williamsburg, VA 23187, USA; eaking@email.wm.edu (E.A.K.); empeairs@email.wm.edu (E.M.P.); dmuthappa@email.wm.edu (D.M.U.); jkvilla@email.wm.edu (J.K.V.); cmgoff@email.wm.edu (C.M.G.); nkburrow@email.wm.edu (N.K.B.); rtdeitch@email.wm.edu (R.T.D.); akmartin@email.wm.edu (A.K.M.)

**Keywords:** non-canonical amino acid, photoactivation, protein methylation

## Abstract

Protein methyltransferases are vital to the epigenetic modification of gene expression. Thus, obtaining a better understanding of and control over the regulation of these crucial proteins has significant implications for the study and treatment of numerous diseases. One ideal mechanism of protein regulation is the specific installation of a photolabile-protecting group through the use of photocaged non-canonical amino acids. Consequently, PRMT1 was caged at a key tyrosine residue with a nitrobenzyl-protected Schultz amino acid to modulate protein function. Subsequent irradiation with UV light removes the caging group and restores normal methyltransferase activity, facilitating the spatial and temporal control of PRMT1 activity. Ultimately, this caged PRMT1 affords the ability to better understand the protein’s mechanism of action and potentially regulate the epigenetic impacts of this vital protein.

## 1. Introduction

Post-translational modifications greatly expand the abilities of enzymes, depending on the chemical properties or conformational changes caused by the newly introduced functionality [1]. Methylation is a common post-translational modification, found ubiquitously in nature, and has been demonstrated to have a diverse range of effects [1,2,3,4]. Protein methyltransferases catalyze the methylation of proteins at the positively charged amino acids’ lysine and arginine by transferring a methyl group from the cofactor *s*-adenosyl-*l*-methionine (SAM) [2,3]. This methylation is especially prevalent in the modification of gene regulation via protein/histone arginine methyltransferases (PRMTs) [1,2,3,4,5,6]. Histone methylation is the most common form of the epigenetic modification of chromatin and primarily determines which sections of the genome are transcriptionally active [6]. Chromatin methylation patterns not only affect gene expression, mitosis, genome stability and DNA repair mechanisms, but are also relevant in disease pathogenesis. Specifically, cancer has been significantly associated with the aberrant methylation of histones and DNA Thus, small-molecule drug development is currently of interest when investigating the modulation of histone methyltransferases for potential therapeutics or chemical probe development [7,8]. The PRMT family of methyltransferases is responsible for the regulation of a variety of cellular mechanisms, such as protein localization, cell fate, and cell signaling [5,6,9,10].

Currently, there are nine known PRMTs in humans, which are all highly conserved, particularly within their catalytic domains. PRMT1 accounts for over 85% of all PRMT activity, although the activity and specificity of all PRMTs are not well documented [11]. PRMT1 is detectible in all tissues, found in all eukaryotes, and closely related to neuronal development [11]. Structurally, the most common splice variant of the PRMT1 protein monomer has 353 amino acids and four core parts: the *n*-terminal domain, the SAM-binding domain, the unique PRMT family β barrel domain, and the dimerization arm [12]. Due to their role and structural features, PRMTs have emerged as attractive anticancer targets and epigenetic regulators to prevent disease genesis. The selective inhibition of PRMT1 is a potential way of helping to prevent or reverse the deleterious effects of methylation misregulation. Rather than knocking out an entire gene, modulation of PRMT1 activity could prevent undesirable methylation while preserving the vital activities of PRMT1 [13]. Moreover, exerting a precise control over its function can lead to a better understanding of its regulation and biological activity. Previous efforts to inhibit PRMTs have focused on non-specific SAM analogs, small-molecule inhibitors, or indirect inhibitors that affect upstream targets of PRMT [14,15,16]. Additionally, previous studies of PRMT1 have involved the incorporation of a phosphotyrosine non-canonical amino acid mimic, *p*-carboxymethyl-l-phenylalanine (*p*CmF), to determine the effect of phosphorylation on PRMT1 activity and better understand the mechanisms of regulation [9].

**Figure 1:**
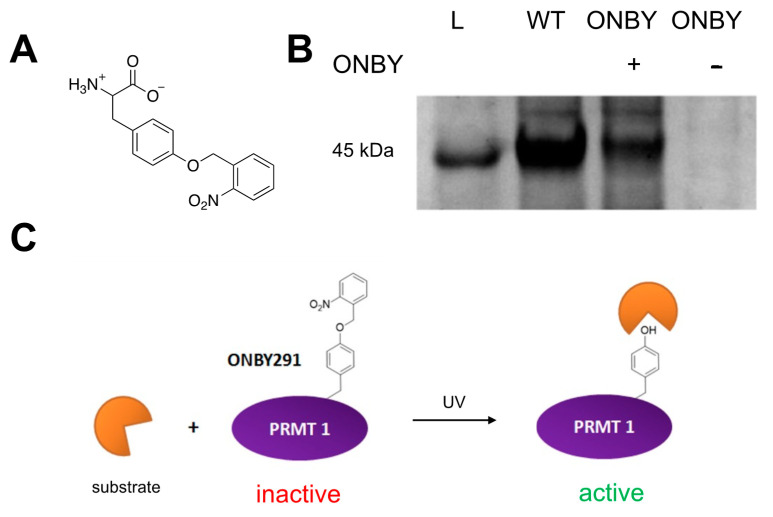
Photochemical regulation of PRMT1 activity. (**A**) Structure of *o*-nitrobenzyltyrosine (ONBY) non-canonical amino acid. (**B**) SDS-PAGE of protein expressions: L = ladder; WT = wild-type protein harboring the natural tyrosine at residue 291; ONBY + = PRMT1 expression containing the TAG codon at residue 291, grown in the presence of ONBY; ONBY − = PRMT1 expression containing the TAG codon at residue 291, grown in the absence of ONBY. Only purified protein expressed in samples containing both the aaRS/tRNA pair and the ONBY non-canonical amino acid is detected. (**C**) Proposed method of PRMT1 photoregulation. With the ONBY residue incorporated at key residue 291, PRMT1 remains inactive. However, in the presence of UV irradiation, the caging group is removed, restoring the natural tyrosine residue and activating the enzyme.

As proteins are typically regulated by complex and precise mechanisms, the exogenous control of protein function is a desirable and powerful tool when studying the mechanism, function, and regulation of proteins [17,18,19,20,21,22,23]. An ideal external stimulus for these mechanisms is light because it is easily tunable and highly defined by its wavelength and intensity. It can also be toggled on and off with high spatiotemporal resolution [21,24]. Light-removable protecting groups, called “caging groups”, are a common mechanism of regulation. These are typically aromatic moieties, which are installed on small-molecule effectors, or even directly on amino acids in the protein of interest. The caging group is positioned based on the active site and native conformation, so that one group is enough to inhibit protein activity [18]. The *ortho*-nitrobenzyl moiety is a well-defined example of such a group, which is employed in proteins such as caged tyrosine or lysine amino acids (ONBY or ONBK; Figure 1A). Brief irradiation with UV light photolytically cleaves the nitrobenzyl group to produce a nitroso-aldehyde and the original amino acid [25]. These have been used in cells to study DNA and RNA, kinases, ion channels, and other enzymes [18,26,27,28,29,30,31,32,33]. While other caging groups with more ideal photochemical properties are available, for this proof-of-concept experiment, the ONBY non-canonical amino acid was selected for its low cost, ease of synthesis, previously established aaRS/tRNA pair, and ability to decage at 365 nm (although this is not the optimal wavelength). Once photoregulation is ascertained to be possible, more ideal caging groups can be translated to cellular and organismal studies.

Traditionally, non-specific reactions of surface amino acids with photolabile reagents have been used to prepare caged proteins [18]. However, a more efficient way to site-specifically incorporate a photocaged amino acid is through genetic code expansion technology [17,28,32,34,35]. This process requires an orthogonal aaRS/tRNA pair to recognize and introduce the amino acid, in a site-specific fashion, at the amber stop codon [36,37,38]. Specific aaRS/tRNA pairs have been evolved to recognize and incorporate photoreactive non-canonical amino acids (ncAAs), including ONBY [34]. Incorporation of the ONBY ncAA in place of a tyrosine can impair or completely inactivate protein function, as the bulky nitrobenzyl group is capable of masking the key hydrogen-bonding interactions and the phosphorylation potential that are present in the wild-type tyrosine residue. This has been well-established in GFP at residue Tyr66, in which substitution with ONBY quenches fluorescence, which was restored post-irradiation [39]. This ncAA was also effective at regulating Cre recombinase via incorporation at a key catalytic residue [19].

A similar mechanism may be used to control PRMT1 activity. Residue Tyr291 has been determined to be important for protein activity, as phosphorylation at this site limits enzymatic activity and alters substrate binding (Figure 1B) [9]. Therefore, we sought to utilize site-specifically incorporated ONBY at Tyr291 to spatially and temporally control PRMT1 activity without the need for small-molecule effectors.

## 2. Results and Discussion

### 2.1. PRMT1 Expression

In order to prepare the caged PRMT1, ONBY was synthesized according to previously reported synthetic protocol under light-free conditions (see Supplementary Material). The *n*- and *c*- termini were subsequently deprotected to yield the ONBY ncAA for genetic incorporation (Figure 1A) [34]. Site-specific incorporation of the ncAA into PRMT1 was then attempted using a plasmid containing an evolved orthogonal aaRs/tRNA pair (pEVOL-ONBY), which was co-expressed in *E. coli* with a PRMT1 protein plasmid (pET-PRMT1-TAG291), harboring the TAG codon at residue 291. Previous work in our lab suggesting that the toxicity of the nitrobenzyl group towards *E. coli* necessitated the use of a decreased concentration (0.5 mM) of ONBY from standard ncAA incorporation expressions. PRMT1 wild-type was also expressed as a control to assess normal PRMT1 activity. Gratifyingly, mutant protein expression was confirmed in the presence of ncAA, but not in the absence of ncAA, by SDS-PAGE (Figure 1B). It is likely that truncated PRMT1 was expressed in the absence of ncAA; however, the truncated product lacks the 6xHis purification tag and is lost during purification. Wild-type protein was purified to a concentration of 2.6 mg/mL and ONBY-incorporated PRMT1 was purified to a concentration of 1.4 mg/mL. The two proteins were expressed greater than 90% purity based on SDS-PAGE and at the molecular weight predicted by ESI-MS, with the WT PRMT1 exhibiting a mass of 40,430 Da and the ONBY-PRMT1 having a mass of 40,566 Da. Both PRMT1 samples were normalized to a final concentration of 0.50 mg/mL for analysis of activity. Importantly, all steps of expression and purification were conducted in light-free conditions, and all steps of purification were conducted on ice to minimize protein degradation.

### 2.2. PRMT1 Activity Assay

Following the successful incorporation of ONBY, PRMT1 mutant and wild-type proteins were assessed for relative activity using a commercially available methyltransferase assay kit (G Biosciences SAM510). In this assay, as histone H4 is methylated, SAM is converted to *s*-adenosyl-l-homocysteine (SAH), triggering a cascade that results in a colorimetric change that can be quantitated via absorbance measurements. This assay accounts for PRMT1’s slow turnover rate by measuring product formation rather than reactant depletion. An additional enzyme, AdoHcy nucleosidase, converts SAH into *s*-ribosyl-l-homocysteine to remove the feedback inhibition of SAH on PRMT1. The byproduct of this conversion, adenine, is deaminated by adenine deaminase into hypoxanthine, which is rapidly oxidized into urate and hydrogen peroxide. The rate of hydrogen peroxide formation is measured by changes in absorbance at 510 nm, in response to the added colorimetric reagents, and can be used to measure PRMT1 activity.

**Figure 2:**
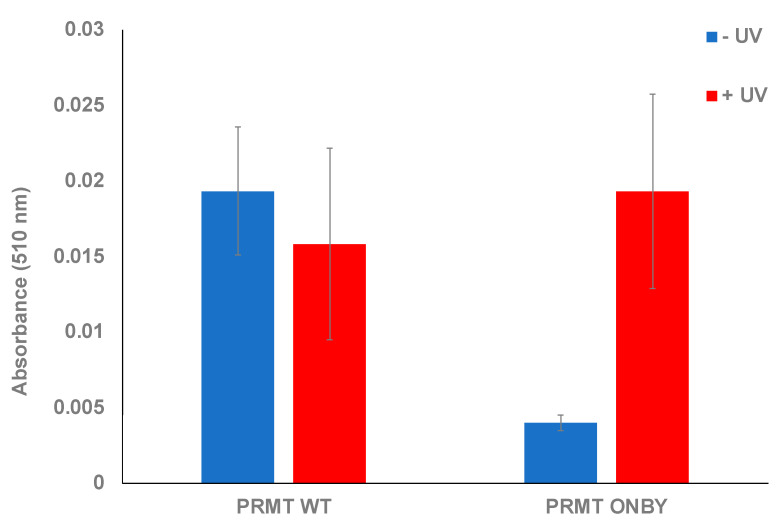
Endpoint assay of caged-PRMT1. Both wild-type protein and ONBY-PRMT1 were assayed in the presence and absence of UV irradiation (2 min at 365 nm). Within the degree of error, no significant effect was observed on the wild-type activity due to light irradiation. However, non-irradiated ONBY-PRMT1 did not possess significant methyltransferase activity unless briefly irradiated, which restored activity to wild-type levels.

This assay was used to confirm the photocaging of PRMT1 and subsequent activation following UV irradiation. Various assays utilizing both the PRMT1 WT, ONBY-PRMT1, and positive and negative controls were performed and monitored at 510 nm after one hour. Initially, the proteins were measured in the absence of UV exposure to ascertain whether the ONBY residue could suppress PRMT1 activity and to establish the baseline activity of PRMT1 WT in the assay conditions. All samples were run in triplicate, and standard deviation was calculated as the measure of data variation. The ONBY-PRMT1 activity was decreased 7-fold in the absence of UV irradiation relative to the wild-type protein, indicating successful inhibition of protein activity (Figure 2). To determine if the inhibition observed from photocaging could be abrogated, a separate plate with the same proteins was irradiated at 365 nm, followed by initiation of the assay. Initial experiments determined that 2 min of irradiation was sufficient for restoration of PRTM1 activity within the range of error (see Supplementary Figure S4). The activity of the irradiated ONBY-PRMT1 was consequently restored to comparable levels to the wild-type protein, suggesting that the spatial and temporal regulation of PRMT1 in this manner is feasible. Moreover, the activity levels of irradiated protein were comparable to the non-irradiated PRMT1 WT, indicating that the UV irradiation did not damage or inactivate the protein at this wavelength and time duration (Figure 2).

### 2.3. Temporal Photoregulation of PRMT1 Function

**Figure 3:**
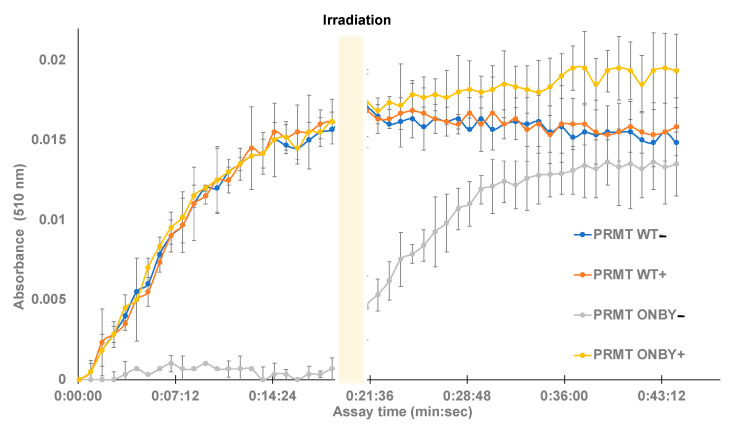
Temporal control of caged-PRMT1. Protein samples were briefly irradiated (+) prior to assay initiation or left initially non-irradiated (−). Methyltransferase assays were conducted in triplicate in a 96-well plate and monitored at 510 nm for the downstream product of enzyme activity. The wild-type (both irradiated and non-irradiated) and the irradiated ONBY-PRMT1 samples exhibited similar kinetic profiles within deviation, while the non-irradiated ONBY-PRMT1 did not harbor enzymatic activity. However, after 20 min, the assay was briefly irradiated, resulting in a gain in function of the previously photocaged protein.

Next, the SAM methyltransferase assay was used to determine whether temporal control of PRMT1 kinetic activity is feasible. PRMT1 and ONBY-PRMT1 were separated into two groups of triplicates, with one group of WT protein and mutant protein irradiated at 365 nm for 2 min (PRMT1 WT + and ONBY-PRMT1 +), and one group not irradiated (PRMT1 WT − and ONBY-PRMT1 −). All of the samples were placed on ice for 5 min and then transferred to the 96-well plate. The assay was initiated in the dark and absorbance was measured every 50 s for a total of 45 min (See Supplementary Material for assay details). The assay included a positive control containing SAH and two negative controls of assay buffer with histone and PRMT1 without histone. After approximately 20 min, the assay was paused, and the entire plate was irradiated at 365 nm for 2 min before resuming measurements for an additional 45 min. A shorter irradiation time was utilized in order to observe the enzymatic activity of decaged PRMT1 prior to saturation, and to minimize any UV damage to the previously irradiated protein samples. Absorbance data were normalized to the negative control and plotted as a function of time (Figure 3). 

Following data analysis, several trends were observed. First, the irradiated and non-irradiated PRMT WT samples had no significant differences between them, suggesting that the irradiation conditions do not cause protein degradation and both samples contained the desired WT activity. Additionally, the negative control of WT PRMT1 with no histone displayed no activity. Moreover, the irradiated ONBY-PRMT1 exhibited similar enzymatic profiles to the wild-type protein, and the non-irradiated ONBY-PRMT1 samples did not appreciably demonstrate enzymatic activity without light activation. Finally, irradiation of the entire plate after 20 min was capable of restoring activity in the ONBY-PRMT1 with similar profiles to the WT. While the substrate conversion did not necessarily reach the same value as the other samples, this may be accounted for by either the somewhat incomplete decaging due to the decreased irradiation time, or the degradation of assay components over time during the initial 20 min of the assay at room temperature. However, these results do demonstrate the potential to temporally regulate PRMT1 activity using light. Moreover, the similar enzymatic profiles demonstrate the ability to effectively decage the enzyme with a brief irradiation and restore enzymatic activity to that of the natural protein.

## 3. Materials and Methods

**General**. pEVOL plasmids were obtained from the laboratory of Prof. Peter Schultz. Assay kits, chemical reagents, and solvents were purchased from either VWR or Sigma-Aldrich and used without further purification. Reactions were conducted under ambient atmosphere with non-distilled solvents. NMR data were acquired on a Varian Gemini 400 MHz instrument. All PRMT proteins were purified according to manufacturer’s protocols using a Qiagen Ni-NTA Quik Spin Kit. Methyltransferase assays were obtained from G Biosciences and stored at −80 °C. All UV irradiation was performed using a hand-held UV lamp (VWR; Catalog number 89131-496, Radnor, PA, USA,) at 365 nm (25 W). The lamp was placed directly on top of the 96-well plate, approximately 2 cm from the sample, for all irradiations. Protein MS data were obtained from an Agilent 6520 Accurate-Mass Quadrupole-Time-of-Flight (Q-TOF) mass spectrometer equipped with an electrospray (ESI) ionization source and liquid chromatography (LC) (Agilent, Santa Clara, CA, USA). Ionization settings were: positive mode; capillary voltage 3500 kV; fragmentor voltage 200 V; drying gas temperature 350 °C. Instrument was set to standard 2 GHz, extended dynamic range and deconvolution was performed by Agilent MassHunter Qualitative Analysis software using the maximum entropy setting. To separate analyte a 2.1 × 150 mm, C8 reverse phase, wide pore (5 μm, 300 Å, Phenomenex, Torrence, CA, USA) column was used with a water (A)/acetonitrile (B) (0.1% formic acid) gradient (2% B for 3 min, followed by a 2–95% B gradient over 15 min, and 95% B for 7 min). 

**Expression of PRMT WT and ONBY-PRMT1**. *Escherichia coli* BL21(DE3) cells were either co-transformed with a pET-PRMT1-TAG-291 plasmid (2.0 μL) and pEVOL-ONBY plasmid (2.0 μL), or only a pET-PRMT1 plasmid, using an Eppendorf electroporator. Cells were then plated on LB-agar plates supplemented with kanamycin (10 mg/mL) and chloramphenicol (34 mg/mL), or kanamycin only (10 mg/mL) for growth, and incubated at 37 °C. After 16 h, a single colony from each plate was selected and used to inoculate 2XYT media (10 mL), supplemented with kanamycin and chloramphenicol, or kanamycin only. The culture was grown to confluence at 37 °C over 16 h. These cultures were used to begin expression cultures in 2XYT media (100 mL) at OD_600_ = 0.1, then incubated at 37 °C until they reached an OD_600_ of between 0.7 and 0.8. At this point, PRMT1-TAG-291 cultures were covered completely in aluminum foil, and cells were induced with 1 M IPTG (100 μL), 20% arabinose (100 μL) and 100 mM ONBY (400 µL), while PRMT1 WT cultures were only induced with 1 M IPTG (100 µL). Induced cells were grown for an additional 16 h at 37 °C, then harvested via centrifugation (10 min, 5000 rpm). The media were decanted, and the cell pellet was stored in a −80 °C freezer for 20 min. PRMT WT protein and ONBY-PRMT mutant protein were then purified using a commercially available Ni-NTA Quik Spin Kit according to manufacturer’s protocol. Protein yield and purity were assessed by SDS-PAGE and assessed spectrophotometrically, using a Nanodrop spectrophotometer. Proteins were buffer-exchanged into PBS, concentrations were measured with a BSA Assay Kit (G Biosciences, St. Louis, MO, USA), and all protein concentrations were diluted to approximately 0.5 mg/mL. Successful expression was confirmed via SDS-PAGE gels stained by Coomassie Blue for protein band visualization. 

**PRMT1 Assay.** SAM510 Methyltransferase Assay provided by GBiosciences (St. Louis, MO, USA) was used according to manufacturer’s protocol to determine the relative activity of the PRMT1 mutants and wild-type either pre- or post-irradiation. A BioTek Synergy HT microplate reader (Winooski, VT, USA) was used to measure the absorbance at 510 nm. Readings were taken every minute for an hour. Either before or after initial reading, the plate was irradiated at 365 nm for 2 min to decage the ONBY group. Absorbance rates were converted to enzyme activity by finding the slope of the change in absorbance, normalization of the data based on the negative control of assay buffer and pure histone with no added protein, and using the Beer–Lambert law and adenine’s molar absorptivity to calculate activity. PRMT1 protein samples were run in triplicate. Substrate for PRMT1 methylation was human recombinant histone 4 (1 mg/mL) from New England Biolabs (Ipswitch, MA, USA). Negative controls were made by excluding histone or the PRMT1 protein. Positive control of AdoHcy was provided in the kit and diluted (1:10) (5 μL of positive control in 45 μL of assay buffer). 14 μL of PRMT1 WT or ONBY (0.5 mg/mL) protein was added to each well (or 14 μL of buffer for the negative control). H4 substrate (1 μL) was then added to give a total volume of 15 μL/well. For the positive control, 5 μL was diluted into 10 μL of buffer. Following sample preparation, the SAM master mix was prepared by adding 3.3 mL of buffer, 100 μL SAM (lyophilized SAM dissolved in 20 mM HCl) and 200 μL of the kit’s enzyme mix (Table S1). Immediately before initiation of the assay, 100 μL of the master mix was added to each well and the plate was placed in the plate reader to immediately begin measuring absorbance. Absorbance measurements were taken every minute for an hour at 37 °C. For temporal studies, the plate was irradiated after 20 min with UV light (365 nm) for 2 min to decage ONBY. Absorbance measurements were then taken every minute for an additional 20 min.

## 4. Conclusions

The use of ncAAs allows for site-specific incorporation, which provides a sophisticated level of control of protein function. PRMTs can act on arginines within a wide range of protein types, indicating their critical function in homeostatic maintenance, but their regulation has been difficult to precisely achieve. The highly specific and tunable exogenous control of human PRMT1 could be important in modulating epigenetic factors and post-translational modifications that result in changes in gene expression, particularly with the application to control histone methylation in disease states such as cancer. 

In this study, the photochemical caging of PRMT1 was attempted and achieved by incorporating the non-canonical amino acid ONBY at the key residue Tyr291. Methyltransferase activity of the caged mutant was analyzed using absorption as compared to the PRMT1 wild-type protein. In both endpoint and kinetic analysis of the caged PRMT1, the activity could be modulated with UV irradiation. These results point towards the successful inhibition of PRMT1 activity through the site-specific incorporation of a single photocaging amino acid. Consequently, the need for small-molecule effectors or complex protein–protein interactions can be eliminated, facilitating more direct studies of the biochemical role of this protein and the misregulation of its activity. The inhibition is easily modulated with UV irradiation, conferring a higher degree of temporal specificity and tunability to this inhibition technique as compared to typical PRMT inhibition methods [8]. In addition to these benefits, PRMT1 can potentially be spatially modulated within the same organism. For example, tissues that require methyltransferase activity would have active PRMT1 upon UV irradiation, while tissues with overactive or abhorrent methyltransferase activity would have no PRMT1 activity. Moreover, PRMT1 activity could be induced at times when it is normally inactive in cells to identify the key substrates and regulatory pathways. However, further investigation into the temperature and time stability of the caged PRMT1, as well as an analysis of specific PRMT activity and protein concentration effects, is necessary, and will be completed in future work. Thus, this study represents a promising method of photo-modulation that can be extended to other proteins for future application in therapeutics and diagnostics. 

## Data Availability

Data is available by request to the authors.

## Supplementary Materials

The supplementary materials are available online.

## Sample Availability

Samples of the compounds and proteins are available from the authors.
