# Supplementary material for: Photoregulation of PRMT-1 Using a Photolabile Non-Canonical Amino Acid
Source: Molecules. 2021 Aug 21;26(16):5072. doi: 10.3390/molecules26165072 (PMC8398576; doi:10.3390/molecules26165072)
Supplement: Supplementary file 1 [file molecules-26-05072-s001.zip › molecules-1321987-supplementary.pdf]

Supplementary Material

Photoregulation of PRMT-1 using a photolabile non-canonical amino acid

Elizabeth A. King,[†] Emily M. Peairs,[†], Diya M. Uthappa[†], Jordan K. Villa, Cameron M. Goff, Naya K. Burrow, Rebecca T. Deitch, Anna K. Martin, and Douglas D. Young[*]

Experimental

Synthesis of O-(2-Nitrobenzyl)-L-tyrosine.

Scheme S1.

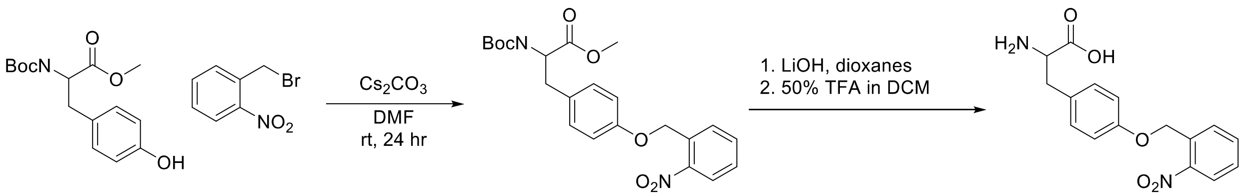

A solution of Boc-Tyrosine-OMe (1.00 g, 1 eq, 3.4 mmol) in DMF (10 mL) was prepared in a foil-covered, flame-dried vial. To this solution, cesium carbonate (3.27 g, 3 eq, 10 mmol) was added and the reaction stirred at room temperature for 10 minutes. Then, 2-nitrobenzylbromide (0.60g, 1.5 eq, 5.0 mmol) was added, and the reaction was stirred overnight at room temperature, then filtered into a foil-covered round-bottom flask. The reaction was extracted using DCM and water (3 x 20 mL each) and the organic layer was dried with MgSO4. Solvent was removed *in vacuo*. The crude product was purified on a foil-covered silica gel column using hexanes:EtOAc (3:1). Pure fractions were combined and concentrated to afford the desired protected product as a brown oil (0.613 g, 1.43 mmol, 42% yield).

To remove the methyl protecting group, a 1:1 LiOH/Dioxane solution (3 mL) was added to the product on ice and stirred for 2 hours at room temperature. The dioxane was then removed in vacuo, the aqueous solution was cooled on ice, and 6 M HCl was added dropwise to the solution until a pH of 4 was achieved. The reaction was extracted and washed with water and EtOAc, and the organic layer was dried over $MgSO_4$ and concentrated in vacuo. To remove the tert-butyloxy protecting group, the yellow oil was resuspended in 50% TFA solution (2 mL TFA/2 mL DCM) on ice and allowed to warm to room temperature for 1 hour. The solvent was then removed in vacuo, and the product was obtained as white solid (408 mg, 90%). NMR and MS data was consistent with previously published results.[1] ESI-MS: $m/z_{expected}$: 317.11; $m/z_{observed}$: 317.15 ^{1}H NMR (400 MHz; d-MeOH): ∂ 8.06 (d, J=7.2, 1H), 7.78 (d, J=7.2, 1H), 7.68 (t, J=6.5, 1H), 7.52 (t, J=6.5, 1H), 7.20 (d, J=6.7, 2H), 6.93 (d, J=6.6, 2H), 5.29 (s, 2H), 4.17 (m, 1H), 3.64 (s, 1H), 3.20 (m, 1H), 1.99 (s, 1H), 1.15 (m, 1H).

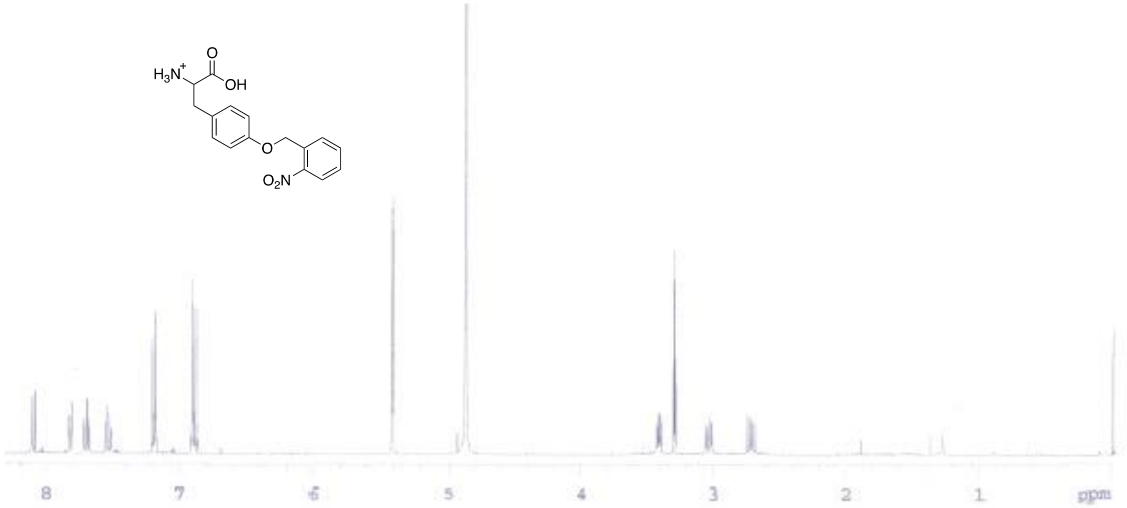

Figure S1. ^{1}H NMR of ONBY non-canonical amino acid.

```
MEVSCGQAESSEKPNAEDMTSKDYYFDSYAHFGIHEEMLKDEVRTLTYRNSMFHNRHLFKDKVVLDVGSGTGILCMF
AAKAGARKVIGIECSSISDYAVKIVKANKLDHVVTIIKGKVEEVELPVEKVDIIISEWMGYCLFYESMLNTVLHARD
KWLAPDGLIFPDRATLYVTAIEDRQYKDXKIHWWENVYGFDMSCIKDVAIKEPLVDVVDPKQLVTNACLIKEVDIYT
VKVEDLTFTSPFCLQVKRNDYVHALVAYFNIEFTRCHKRTGFSTSPESPYTHWKQTVFYMEDYLTVKTGEEIFGTIG
MRPNAKNNRDLDFTIDLDFKGQLCELSCSTDYRMR
```

Figure S2. Protein Sequence of PRMT1. The non-canonical amino acid site is highlighted in yellow.

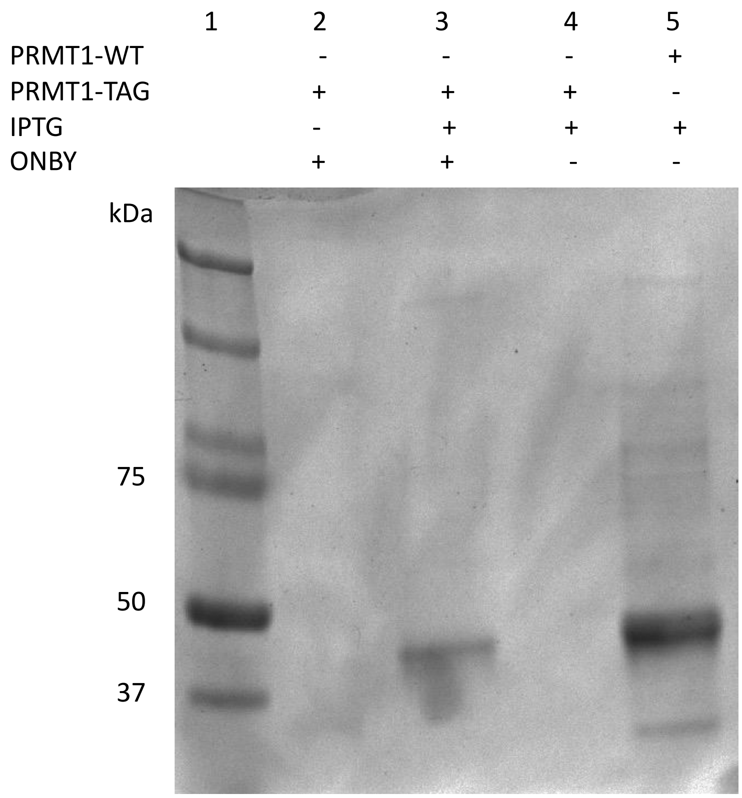

Figure S3. Full length SDS-PAGE of PRMT1 expressions. Lane 1: Ladder; Lane 2: PRMT1-ONBY expression in the absence of IPTG; Lane 3: PRMT1-ONBY expression in the presence of ONBY and IPTG; Lane 4: PRMT1-ONBY expression in the presence of IPTG and absence of ONBY; Lane 5: PRMT1-WT expression.

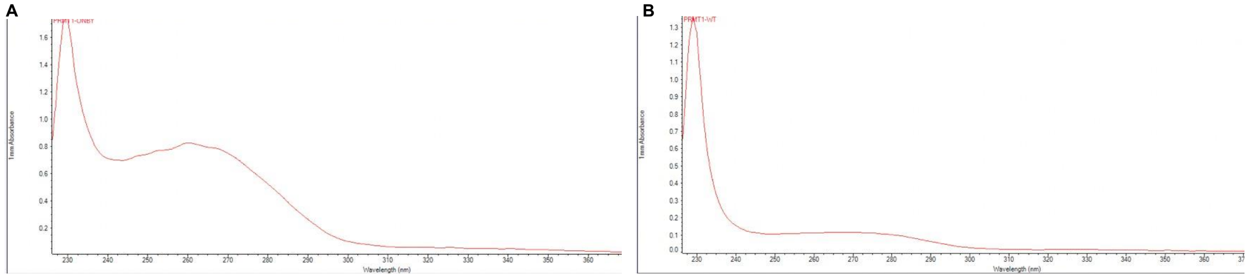

Figure S4. UV-Vis analysis of PRMT1-ONBY (A) and PRMT1-WT (B) samples.

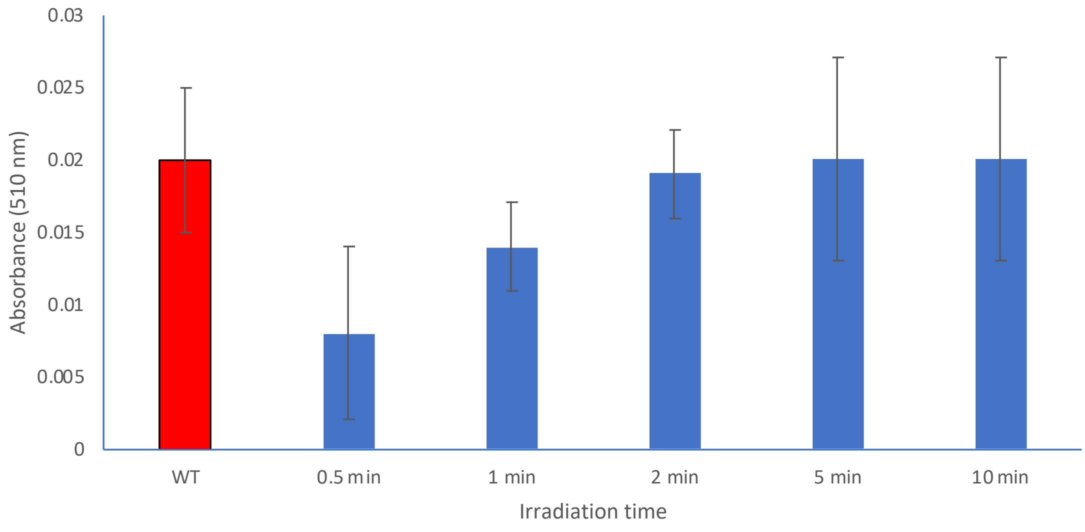

Figure S5. PRMT1 decaging endpoint assay. The PRMT1-ONBY was irradiated with a handheld UV lamp at 365 nm for various times and then subjected to the methylation assay. Within error restoration to wild-type activity could be achieved after 2 min of 365 nm irradiation.

Table S1: All conditions initially tested in the SAM510 Methyltransferase Assay. A “--” denotes no addition of the corresponding component. Each condition was run in triplicate and all samples were run on one 96-well plate. Irradiation was conducted prior to assay. All proteins were used at a concentration of ~0.5 mg/mL.

**Conditions Tested**							
	**1**	**2**	**3**	**4**	**5**	**6**	**7**
Histone H4	1 µL	1 µL	1 µL	1 µL	--	1 µL	--
PRMT1 WT	10 µL	10 µL	--	--	--	--	10 µL
ONBY-PRMT1	--	--	10 µL	10 µL	--	--	--
Positive Control	--	--	--	--	5 µL	--	--
Buffer	4 µL	4 µL	4 µL	4 µL	10 µL	14 µL	5 µL
Irradiation	365 nm 2 min.	None	365 nm 2 min.	None	None	None	None

References

[1] Dieters A, Groff D, Ryu Y, Xie J, Schultz PG. A genetically encoded photocaged tyrosine. *Angew Chem Int Ed.* 2006;45(17), 2728-2731.
[2] G-Biosciences, St. Louis, MO. SAM510: SAM Methyltransferase Assay Kit (catalog no. 786-430).
